# Describing adolescents with disabilities’ experiences of COVID-19 and other humanitarian emergencies in low- and middle-income countries: a scoping review

**DOI:** 10.1080/16549716.2022.2107350

**Published:** 2022-09-06

**Authors:** Brigitte Rohwerder, Sara Wong, Shraddha Pokharel, Dipesh Khadka, Niraj Poudyal, Sagar Prasai, Nir Shrestha, Mary Wickenden, Joanna Morrison

**Affiliations:** a Institute of Development Studies, University of Sussex, Brighton, England; b UCL Institute for Global Health, London, England; c Diverse Patterns, Battisputali, Kathmandu, Nepal; d School of Arts, Kathmandu University, Lalitpur, Nepal

**Keywords:** Youth, inclusion, disabled, conflict, natural disaster

## Abstract

**Background:**

The COVID-19 pandemic and other humanitarian emergencies exacerbate pre-existing inequalities faced by people with disabilities. They experience worse access to health, education, and social services, and increased violence in comparison with people without disabilities. Adolescents with disabilities are amongst those most severely affected in these situations. Using participatory research methods with adolescents can be more effective than other methods but may be challenging in such emergency contexts.

**Objectives:**

We conducted a scoping review to: 1) describe the literature and methods used in peer-reviewed and grey literature on adolescents (aged ten to nineteen) with disabilities’ experience of COVID-19 and other humanitarian emergencies in low- and middle-income countries, and 2) identify research gaps and make recommendations for future research.

**Methods:**

The review followed a protocol developed using PRISMA guidelines and the Arksey and O’Malley framework. We searched grey and peer-reviewed literature between 2011 and 2021.

**Results:**

Thirty studies were included. Twelve were peer-reviewed, and of those seven used participatory methods. Humanitarian emergencies had adverse effects on adolescents with disabilities across health, education, livelihoods, social protection, and community participation domains. Surprisingly few studies collected data directly with adolescents with disabilities. Twenty-three studies combined data from non-disabled children, caregivers, and disabled adults which made it challenging to understand adolescents with disabilities’ unique experience.

**Conclusions:**

Our review highlights both the scarcity of literature and the importance of conducting research with adolescents with disabilities in humanitarian contexts. Despite challenges, our review shows that it has been possible to conduct research with adolescents with disabilities to explore their experiences of humanitarian emergencies, and that these experiences were different from those of non-disabled adolescents. There is a need to disaggregate findings and support the implementation and reporting of rigorous research methods. Capacity development through partnerships between non-governmental organisations and researchers may improve reporting of methods.

## Background

There is increasing evidence that humanitarian emergencies, such as the COVID-19 pandemic, have worsened pre-existing inequalities, particularly affecting those already marginalised by disability, age, and low socio-economic status [1–3]. For example, people with disabilities routinely experience discrimination. This means that they are often denied equitable access to resources, services and opportunities for personal development, which results in lower levels of education and higher levels of poverty than in people without disabilities [4,5] During humanitarian disasters, rapid assessments have shown that people with disabilities experienced worse access to health, education, and social services, and increased violence in comparison with people without disabilities [2,3]. These inequalities are driven by pre-existing socio-economic and political processes that structure hierarchical power relations and stratify society on the basis of gender, disability, age etc [1]. These inequalities mean that adolescents (aged ten to nineteen) with disabilities are amongst those most severely affected by humanitarian emergencies such as the COVID-19 pandemic [3,6]. Context-specific research is therefore necessary to capture the diverse experience of those with intersecting vulnerabilities to implement informed humanitarian responses.

Research about COVID-19 and disability, and humanitarian emergencies and disability, often focuses on measurable health outcomes without analysing the complexity of intersectional experience [7] and there has been little research from low- and middle income countries (LMICs) [8,9]. We sought to describe: 1) the literature on the experience of adolescents with disabilities during large-scale humanitarian emergencies in LMICs (including the COVID-19 pandemic), and 2) the methods used in these studies. We were particularly interested in the extent to which the studies engaged adolescents themselves as participants in the research process, as this can enable inclusive research. Their participation can help to focus on issues of relevance and importance to adolescents with disabilities and enable the development of sensitive tools and research methods to improve the quality and authenticity of data [10–12], but can be challenging to implement in humanitarian contexts.

## Methods

We followed a protocol that was developed a priori [13] using PRISMA guidelines [14] and followed recommendations by Levac et al. [15] on the Arksey and O’Malley framework [16]. We asked: 1) how have adolescents with disabilities experienced large-scale humanitarian emergencies (including disease outbreaks (e.g. COVID-19), natural disasters (e.g. earthquakes), and conflict) in LMICs, and 2) what research methods and tools have been used to research these experiences?

### Search strategy

**Figure 1.**
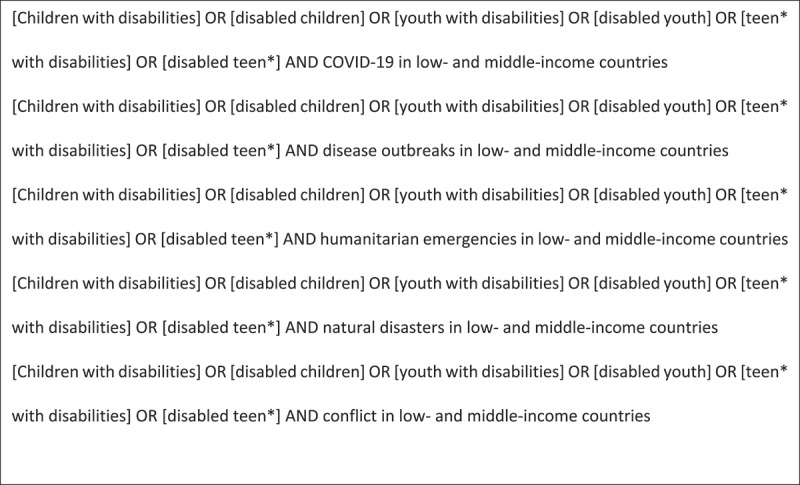
Search terms.

We sought both peer-reviewed and grey literature in English between 1 May 2011 and 30 May 2021. We searched Scopus, Web of Science, ASSIA, Jstor, Source, Google Scholar, Academia.edu and ResearchGate to access literature across health, education, livelihoods, social protection, and community participation domains. We limited our review of Google to the first ten pages. We searched the websites of United Nations High Commissioner for Refugees (UNHCR), World Health Organisation (WHO), United Nations Children’s Fund (UNICEF), Humanity and Inclusion (HI), CBM International, International Disability Alliance (IDA), and Women’s Refugee Commission (WRC). For websites without a rigorous search function, we used the search website function in google – site: [website] ‘[search term]’ and limited our search to the first ten pages. We also sourced literature through personal contacts and our national advisory committee. We used search terms related to youth, disability, humanitarian emergencies, low-and middle-income countries and COVID-19 (Figure 1). Four reviewers (BR, DK, SW, and SP) searched databases.

### Study selection

**Figure 2.**
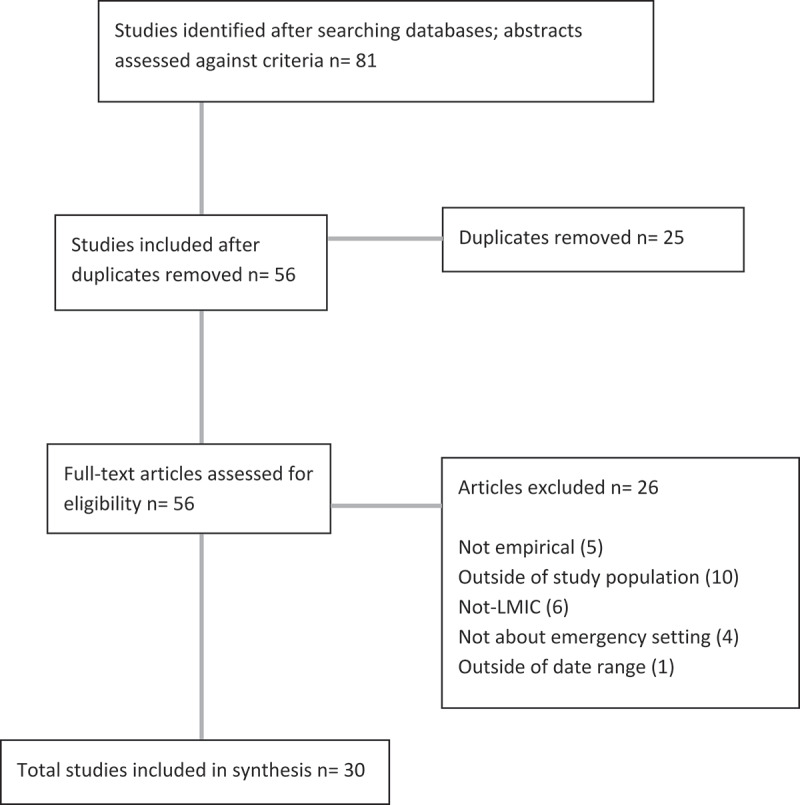
Study selection.

Abstracts and titles were screened for inclusion by four reviewers. As stated in our protocol, we did not assess quality of the studies in our review because we anticipated that it would be challenging to apply criteria to grey literature which may not report methods consistently. Studies were included if they reported on empirical data about adolescents with disabilities between the ages of ten and nineteen from LMICs as classified by the World Bank [17]. We also included literature about children from a wider age range if adolescents with disabilities aged ten to nineteen were included. We excluded research about adolescents with disabilities who were disabled by or after the humanitarian emergency event, or by COVID-19. Thirty studies were included (Figure 2).

### Data extraction

Five researchers charted key items of information from the literature onto a shared excel document. We charted publication year, literature type (grey or peer-reviewed), study population (age group and number of participants), methodology (qualitative, quantitative, or mixed methods), methods and sampling (including whether they were participatory or not), and study location (rural, urban, or other). Initially, reviewers extracted data from the same five publications to check for consistency and develop thematic categories to chart the findings. We reported findings within themes of: health and wellbeing (including mental health, rehabilitation, and violence and abuse); education; livelihoods and social protection; community (how emergencies affect the ability of disabled children to participate in their community, including social care, support networks, transport and infrastructure, social life, and stigma), and inclusion in emergency responses (access to services, information, needs assessments and data disaggregation, disaster planning) [18]. Uncertainties were discussed in weekly meetings. References were uploaded to Zotero (Table 1).

**Table 1.** Included studies.

Study	Disaster type	Literature type	Methodology and data collection	Study population	Location	Findings included
Amnesty International (AI). (2019).	Conflict/displacement	Grey literature	Qualitative – in person	Adolescents with disabilities and their parents/caregivers	Yemen	HealthEducationLivelihoods and social protectionCommunityInclusion in emergency response
Bakhshi, P., Babulal, G., & Trani, J.-F. (2018).	Conflict/displacement	Peer reviewed	Quantitative – in person	Adolescents with disabilities and their parents/caregivers	Darfur, Sudan	HealthEducationLivelihoods and social protection
Bista, S. B., & Sharma, S. (2019).	Natural disaster	Peer reviewed	Qualitative – in person	Adolescents with disabilities	Nepal	Health
Brennan, C. S. (2020).	COVID-19	Grey literature	Mixed methods – virtually	Adolescents with disabilities and their parents/caregivers (focused on people with disabilities generally)	Africa, Asia, Central America, Eastern Europe, European Union, Middle East, North America, Oceania, South America, and the Caribbean (reviewed findings from LMICs)	HealthInclusion in emergency response
Danquah, L., Polack, S., Brus, A., Mactaggart, I., Houdon, C. P., Senia, P., Gallien, P., & Kuper, H. (2015).	Natural disaster	Peer reviewed	Quantitative – in person	Adolescents with disabilities	Haiti	HealthEducationCommunity
Deitch, J., & Gambir, K. (2021).	COVID-19	Grey literature	Qualitative – in person – participatory	Adolescents with disabilities	Cox’s Bazaar, Bangladesh	Health
Emirie, G., Iyasu, A., Gezahegne, K., Jones, N., Presler-Marshall, E., Workneh, F., & Yadete, W. (2020).	COVID-19	Grey literature	Qualitative – phone	Adolescents with disabilities and their parents/caregivers	Ethiopia	HealthEducationLivelihoods and social protectionCommunity
HI Nepal. (2020).	COVID-19	Grey literature	Mixed methods – phone	Parents, caregivers, support staff and/or teachers	Nepal	Education
Holmes, R., Samuels, F., & Ghimire, A. (2018).	Natural disaster	Grey literature	Mixed methods – in person	Adolescents with disabilities and their parents/caregivers	Nepal	HealthLivelihoods and social protection
Holmes, R., Samuels, F., Ghimire, A., & Thewissen, S. (2018).	Natural disaster	Grey literature	Mixed methods – in person	Adolescents with disabilities and their parents/caregivers	Nepal	HealthLivelihoods and social protection
Humanity & Inclusion. (2020).	COVID-19	Grey literature	Mixed methods – in person	(focused on people with disabilities generally)	Multi-country(Africa, Asia, Middle East)	HealthLivelihoods and social protectionCommunityInclusion in emergency response
Jones, N., Guglielmi, S., Małachowska, A., Hamad, B. A., Hamad, S. A., Hamra, E. A., Alam, F., Alheiwidi, S., Alabbadi, T., Al-Redaisy, N., Amaireh, W., Amdeselassie, T., Banioweda, K., Diab, R., Gebeyehu, Y., Gezahegne, K., Iyasu, A., Qandeel, A., Sultan, M., … Workneh, F. (2021).	COVID-19	Grey literature	Qualitative – phone	Adolescents with disabilities	Multi-country (Bangladesh, Ethiopia, Jordan and Palestine)	HealthEducationCommunityInclusion in emergency response
Jones, N., Małachowska, A., Guglielmi, S., Alam, F., Hamad, B. A., Alheiwidi, S., & Yadete, W. (2020).	COVID-19	Grey literature	Mixed methods – virtual – participatory	Adolescents with disabilities	Multi-country (Bangladesh, Ethiopia, Jordan and Palestine)	HealthEducationLivelihoods and social protectionCommunity
Jones, N., Presler-Marshall, E., & Stavropoulou, M. (2018).	Conflict/displacement	Grey literature	Mixed methods – in person – participatory	Adolescents with disabilities and their parents/caregivers	Multi-country (Bangladesh,Ethiopia, Jordan and Palestine.)	HealthEducationLivelihoods and social protectionCommunity
Mbazzi, F. B., Nalugya, R., Kawesa, E., Nimusiima, C., King, R., Hove, G. van, & Seeley, J. (2021).	COVID-19	Peer reviewed	Qualitative – phone	Adolescents with disabilities and their parents/caregivers	Uganda	HealthEducationLivelihoods and social protectionCommunityInclusion in emergency response
Mbukwa-Ngwira, J., Johansson, S. T., Singal, N., Umar, E., Lynch, P., & Chatha, G. (2021)	COVID-19	Greyliterature – blog	Quantitative – phone	Parents, caregivers, support staff and/or teachers	Malawi	HealthEducation
McClain-Nhlapo, C., Singh, R. K., Martin, A., Alasuutari, H., Baboo, N., Cameron, S., Hayes, A., Johnstone, C., Maladwala, A., McGeown, J., Richler, D., & Singal, N. (2020).	COVID-19	Peer reviewed	Mixed methods – virtually	Adolescents with disabilities and their parents/caregivers	Multi-country (East Asia and Pacific;Europe and Central Asia; Latin America and the Caribbean;Middle East and North Africa; North America; South Asia;Sub-Saharan Africa)	HealthEducationLivelihoods and social protection
Orsander, M., Mendoza, P., Burgess, M., & Arlini, S. M. (2020).	COVID-19	Grey literature	Mixed methods – in person and virtual	Adolescents with disabilities and their parents/caregivers	Multi-country (46 countries in Asia; Eastern and Southern Africa; West and Central Africa; Middle East and Europe; Latin America and the Caribbean; North America; Pacific)	HealthEducationLivelihoods and social protectionCommunityInclusion in emergency response
Pearce, E., Paik, K., & Robles, O. J. (2016).	Conflict/displacement	Peer reviewed	Qualitative – in person – participatory	Adolescents with disabilities and their parents/caregivers	Multi-country (South Sudan, Ethiopia, Burundi, Tanzania, Uganda, Jordan,Turkey, Egypt, Iraq, and the Northern Caucasus in the Russian Federation)	HealthLivelihoods and social protectionCommunity
Presler-Marshall, E., Jones, N., & Odeh, K. B. (2020).	Conflict/displacement	Peer reviewed	Qualitative – in person – participatory	Adolescents with disabilities and their parents/caregivers	Multi-country (Jordan and the State of Palestine)	HealthEducationLivelihoods and social protectionCommunity
Rohwerder, B., Thompson, S., Shaw, J., Wickenden, M., Kayastha, S., Sigdel, A., Akter, F., & Bosri, R. (2021).	COVID-19	Grey literature	Qualitative – virtual	Parents, caregivers, support staff and/or teachers (focused on people with disabilities generally)	Multi-country (Bangladesh, Kenya, Nepal, Nigeria, Uganda)	Health and wellbeingEducationLivelihoods and social protection
Sharpe, D., Rajabi, M., Chileshe, C., Joseph, S. M., Sesay, I., Williams, J., & Sait, S. (2021).	COVID-19	Peer reviewed	Mixed methods – in person	Adolescents with disabilities	Multi-country (Zambiaand Sierra Leone)	HealthEducationCommunity
Stakeholder Group of Persons with Disabilities. (2020).	COVID-19	Grey literature	Qualitative – virtually	Adolescents with disabilities and their parents/caregivers (focused on people with disabilities generally)	Multi-country (Africa; Asia; Europe and North America; Middle East and North Africa; Latin America)	HealthEducationCommunity
Swabhiman. (2020).	COVID-19	Grey literature	Mixed methods – in person and virtual	Adolescents with disabilities and their parents/caregivers	India	HealthEducationLivelihoods and social protectionInclusion in emergency response
Tanabe, M., Nagujjah, Y., Rimal, N., Bukania, F., & Krause, S. (2015).	Conflict/displacement	Peer reviewed	Qualitative – in person – participatory	Adolescents with disabilities	Multi – country (Kenya, Nepal, and Uganda)	
Tanabe, M., Pearce, E., & Krause, S. K. (2018).	Conflict/displacement	Peer reviewed	Qualitative – in person – participatory	Adolescents with disabilities and their parents/caregivers	Multi – country (Kenya, Nepal, and Uganda)	Health
Tchie, A. E., & Tkacova, K. (2018).	Conflict/displacement	Grey literature	Quantitative – in person	Adolescents with disabilities and their parents/caregivers	Syria	HealthEducation
Trani, J.-F., Fowler, P., Bakhshi, P., & Kumar, P. (2019).	Conflict/displacement	Peer reviewed	Quantitative – in person	Adolescents with disabilities and their parents/caregivers	Afghanistan	Education
UNICEF. (2020).	COVID-19	Grey literature	Mixed methods – virtual	Adolescents with disabilities and their parents/caregivers	Philippines	HealthEducationLivelihoods and social protectionCommunityInclusion in emergency response
Zuurmond, M., Nyapera, V., Mwenda, V., Kisia, J., Rono, H., & Palmer, J. (2016).	Conflict/displacement	Peer reviewed	Qualitative – in person	Parents, caregivers, support staff and/or teachers	Turkana, Kenya	HealthEducationLivelihoods and social protectionCommunityInclusion in emergency response

### Data synthesis

Data were synthesised descriptively and grouped according to literature type, methodology, method, and findings. A relatively large proportion of the literature concerned the pandemic, so we grouped data into COVID-19 related and literature about other humanitarian emergencies for comparative analysis. We discussed our findings and draft implications with our advisory committee.

## Results

### Literature type

Twelve studies were peer-reviewed [19–30] and 18 were grey literature [31–47], including one blog [40].

### Methodology

Thirteen studies used qualitative methods [20,22,25,27,28,30,31,33,34,38,43,44,48], five used quantitative methods [19,21,29,40,46]. Twelve studies used mixed methods [23,26,32,35–37,39,41,42,45,47,49]. Methodological approaches were evenly mixed across the literature about mass disease outbreaks, natural disasters, and displacement/conflict.

Seventeen studies used in-person data collection [19–21,25-31,33,35–37,46,48,49]. All studies conducted in conflict, displacement and natural disaster settings were in-person, while only three of the studies during the COVID-19 pandemic used in-person data collection [26,33,35]. Five studies used phone data collection [22,34,39–41]. Six studies used virtual methods [23,32,38,43,44,47]. Two studies used a mix of in-person and virtual methods [42,45].

Seven studies used participatory methods [25,27,28,33,37,38,48]. All of these studies used a qualitative methodology except one mixed methods study [37]. Of the participatory studies, three engaged only with adolescents with disabilities [27,33,38], and four engaged with adolescents and their caregivers [25,27,37,48]. Participatory methods included mapping [25,27,28,33] storytelling, vignettes, diaries, photography and photo elicitation, visual methods, and peer-to-peer data collection.

### Study population

Four studies collected data only from parents, caregivers, support staff and teachers [30,40,43,50], and seven studies collected data only from adolescents with disabilities [20,21,26,28,33,38,39]. Eighteen studies engaged with adolescents with disabilities and their caregivers [19,22,23,25,27,29,31,32,34,36,37,42,44–49]. There was only one study that did not clearly state the research population [35].

It was often difficult to ascertain whether findings were specific to one age group, and findings were often reported in summary for wide age ranges of children from ten months old and upwards. Some aggregated the experiences of adolescents and adults with disabilities. All the studies using participatory methods focused on adolescents aged ten to nineteen years.

### Study setting

We searched for studies about mass disease outbreaks, but the search only returned seventeen studies about the experiences of adolescents with disabilities during the COVID-19 pandemic. Eight studies focused specifically on the experiences of adolescents with disabilities and their families during the pandemic [22,23,34,40–42,45,47]. Four studies included adolescents with disabilities in their study population of adolescents [26,33,38,39]. The remaining five papers examined experiences of the pandemic among people with disabilities but included the experiences of adolescents with disabilities [32,35,43,44].

Ten studies examined the experience of adolescents with disabilities in conflict and displacement settings prior to the pandemic. Six were in settings of conflict and internal displacement [19,29–31,46,48] and four in conflict and/or refugee settings [25,27,28,37]. The studies in conflict and refugee settings used the same dataset, with one study focused on research methods [28].

Four studies reported on adolescents with disabilities experience of natural disasters. Three were about the 2015 Nepal earthquakes [20,36,51] and one was about the 2010 Haiti earthquake [21]. Two of the Nepal studies used the same dataset [36,51].

### Health and wellbeing

#### Health service access

During COVID-19, routine health services and health communication were suspended or more difficult to access than normal [22,32–35,38,42,47]. Adolescents with disabilities were denied access to healthcare on an equal basis with non-disabled adolescents because of worsened stigma, discrimination, physical barriers, and financial barriers [22,32–35,38,42,47]. In some Asian countries parents were not allowed to accompany their adolescents with disabilities into hospital, which led to poorer care and death [44]. Quarantine and isolation facilities were not adapted for those with disabilities [35].

Health services in conflict and displacement settings were either lacking, of poor quality, or poorly tailored to meet the needs of adolescents with disabilities [31,37]. Adolescents with disabilities in disaster, conflict, and displacement settings faced greater difficulties accessing health care than adolescents without disabilities [20,25,28]. In these settings, adolescents with disabilities often had greater needs and were poorer than non-disabled adolescents [25]. Data from refugee camps in Cox’s Bazaar showed how the pandemic exacerbated challenges to accessing health care, especially for refugee girls with disabilities [33].

#### Specialised treatment, rehabilitation, and medication

During the COVID-19 pandemic, specialised treatment, essential therapies, and rehabilitation were disrupted, and families struggled to get medication for adolescents with disabilities [22,38,40,44,45,47]. In conflict and displacement settings, access to specialist care (such as physiotherapy for adolescents with disabilities to prevent long-term damage) was expensive or not available, despite the significant need [25,30,31,46,48]. High-quality, disability-specific health care was rarely sustained because it was often provided by non-government organizations (NGOs) with small budgets and short funding cycles [25,48]. A lack of regular specialised and non-specialised services resulted in the deterioration of the health and capabilities of adolescents with disabilities, especially girls and those with intellectual disabilities [31,37,47,48].

#### Accessible information about COVID-19 prevention

A lack of accessible information about COVID-19 and prevention measures made it difficult for adolescents with disabilities to apply public health advice [22,23,33,35,47]. They tended to rely on their parents for information [22,42,45,47]. When they were aware of the guidance, it was difficult to follow [33,45]. For those living in poverty, it was hard to afford soap, sanitiser and masks and some adolescents with disabilities had no access to handwashing facilities [23,33,45]. Social distancing was challenging for those who relied on the assistance of others. Adolescents with physical or visual disabilities in some parts of Ethiopia found that people were unwilling to give them assistance. Adolescents with disabilities were also afraid of catching COVID-19 from helpers or on public transport, particularly if mask wearing was not common [34]. In other parts of Ethiopia where there was less adherence to social distancing measures, some adolescents with disabilities were mocked for wearing masks [34].

#### Nutrition impacts

Malnutrition of adolescents with disabilities was of concern in humanitarian emergency settings, particularly amongst girls and those with intellectual disabilities. Poverty, food price increases and inadequately targeted assistance programmes led to poor nutrition and distress [25,37]. In Turkana, Kenya (a complex humanitarian setting), adolescents with disabilities were more likely to be malnourished than their neighbours and siblings [30]. Food insecurity because of the pandemic was also commonly reported [22,23,26,38,42,47].

#### Mental health impacts

In humanitarian emergencies and COVID-19 contexts, the mental health needs of adolescents with disabilities increased. Often, adolescents with intellectual disabilities did not understand the pandemic and were distressed because they were confined at home. This frustration initially resulted in aggressive behaviours which improved over time [43,44]. Lockdowns, school closures, isolation, food insecurity, economic pressures, and disruption to life during the pandemic meant that many adolescents with disabilities were bored, sad, stressed, anxious, and angry, and suicidal [26,38–40,42,45,47]. A multi-country study looking at the differences between adolescents with disabilities and their non-disabled peers found that adolescents with disabilities were more likely to lose sleep, be distressed and engage in aggressive behaviours than children without disabilities [42]. Mental health support services were inaccessible or unavailable to meet these increased needs [26,38,45,47]. Adolescents with disabilities in the conflict context of Darfur, Sudan, had a lower state of psychological wellbeing than non-disabled adolescents, making them more susceptible to mental health disorders [19]. Being confined at home, being dependent on others, or being perceived as a burden to their family had a psychological impact on adolescent girls with disabilities in humanitarian settings [48]. In Nepal, parents reported that adolescents with disabilities were more scared and displayed more challenging behaviour after the earthquake than their non-disabled siblings [36,49].

#### Violence and abuse

Humanitarian emergencies also resulted in increased risks of violence and abuse for adolescents with disabilities. During the pandemic there were often tensions in the home due to lockdowns and economic pressures [38,39]. Some adolescents with disabilities reported violence and abuse from their family, as well as neighbours and strangers online [35,39,42,47]. In conflict-affected humanitarian settings, increased power and dominance over adolescent girls with disabilities and lessened protective systems put them at greater risk of violence and abuse [48]. Stressed family situations created problems for those who were dependent on other family members, and camps or shared accommodation offered little privacy [48]. As a result, adolescents with disabilities, especially girls with disabilities and adolescents with intellectual disabilities, were more likely to experience violence than their peers without disabilities. This had multiple and long-term consequences for their physical and psychosocial wellbeing [28,31,37]. There were reports that girls with disabilities in post-earthquake Nepal experienced increased psychological, physical, and sexual violence in comparison to their pre-earthquake experiences. This violence was experienced in and around temporary shelters [20,36,49]. Adolescents with disabilities also struggled to access protection mechanisms and seek justice [37,48]. During the pandemic in Ethiopia adolescents with disabilities who worked in the street experienced police violence during the enforcement of lockdown regulations [34,38].

### Education

#### Education in humanitarian settings

Many adolescents with disabilities in conflict-affected contexts, especially refugees, were not in school even if they had attended before the conflict began [19,31,46]. They faced additional challenges to accessing education than non-disabled adolescents because there were few accessible schools, and there were security challenges whilst travelling to school [37]. Commonly cited barriers to education were inaccessible classes, poor quality teaching, limited efforts made to accommodate learning needs, and disability stigma [25]. In humanitarian settings, both disabled and non-disabled adolescents were out of school because of poverty and violence from teachers, staff, other children or community members while they were on the way to school [19]. In Yemen, transport to school was expensive, and some adolescents with disabilities were displaced far from specialised schools. Schools in Internally Displaced Persons’ camps were inaccessible and teaching practices were not inclusive [31]. In Afghanistan, girls with disabilities were less likely to access school and be literate compared with boys with disabilities. Children with physical disabilities were more likely to access school and be literate than children with intellectual disabilities [29].

#### Education during COVID-19

During the pandemic many schools and day centres were closed. This meant that adolescents with disabilities missed education, socialisation and stimulation [34,43,47]. Remote learning at home was problematic for adolescents with disabilities and they struggled in comparison to non-disabled adolescents. Remote learning materials were often inaccessible and expensive. It was difficult for them to participate in online learning due to issues with accessibility and the cost or availability of equipment and the internet. Online learning was particularly inaccessible for children with sensory disabilities [34,44,45]. Parents often felt unable to support learning. They found it particularly challenging to support adolescents who had sensory disabilities, and motivation for home schooling waned over time [22,23,26,34,38–42,44,45,47]. Adolescents with disabilities spent little time studying and parents were worried about the detrimental effect of this on their future [22,26,40,41,44,45]. Some parents were very dissatisfied with their disabled children’s remote learning and teachers’ level of engagement [40,41,45].

#### Non-educational school benefits

School closures during the pandemic meant that some adolescents with disabilities missed out on therapies and rehabilitation provided at school. This contributed to their deterioration [40,47]. When schools had feeding programmes, closures increased food insecurity and malnutrition [23,45]. The suspension of school stipends for adolescents with disabilities in Ethiopia also contributed to food insecurity [34]. In the complex humanitarian context of Turkana, Kenya, research showed that adolescents with disabilities missed out on school feeding programmes as they were less likely to be in school [30].

#### Returning to school

Some parents were worried about their children’s return to school as they could not afford school fees, uniforms, and supplies because of the economic effects of the pandemic [22,23,26,40]. Two years after the earthquake in Haiti, adolescents with disabilities, especially girls, were less likely to be enrolled at school than children without disabilities [21].

### Livelihoods and social protection

#### Increased poverty

During the pandemic, costs increased and adolescents with disabilities and their families became poorer than they were before the pandemic. This contributed to their distress, food insecurity, and inability to meet health and education needs [22,34,35,38,42,45,47]. Some parents had to leave work to look after their children who were at home instead of at school, which put additional pressure on the family [43].

In COVID-19 and humanitarian settings, livelihoods were lost, and social protection was disrupted. Research shows that social protection did not increase to align with increased costs [22,23,34,35,38,45]. Families’ financial stress made them less able to provide care to adolescents with disabilities [25]. In Darfur, Sudan and Turkana, Kenya, adolescents with disabilities were more likely to live in female headed households, where the household head was not educated. This increased their poverty risk [19,30]. In Kenya, mothers struggled to pursue livelihood activities alongside caregiving, often without the support of their extended family [30]. As a result, adolescents with disabilities were often perceived to be a burden [30]. In a multi-country study, older adolescents with disabilities experienced barriers to access the skills, credit, assets and opportunities needed to engage in decent and productive livelihoods [37]. During the pandemic, some adolescents with disabilities and their families received support from organisations of persons with disabilities, families, neighbours or remote support from community-based rehabilitation workers [23]. A study from Ethiopia suggests that dependence on this charity was stressful for families because it could be withdrawn [34].

#### Social protection provision

Adolescents with disabilities and their families were often excluded from relief distributions during the pandemic and in conflict-affected settings. Financial assistance was often insufficient [35,45,47]. Presler-Marshall et al. reported that parents of disabled adolescents were forced to choose between food and hygiene needs in conflict-affected settings [25].

In post-earthquake Nepal, there were some delays in accessing social support because government offices were closed, but these were resolved when government offices resumed activities [36,49]. Earthquake-displaced families found it difficult to return home to receive their disability allowance [36,49]. Only some adolescents with disabilities could receive the disability allowance due to difficulties registering for a disability identity card [36,49].

### Community

#### Barriers to participation

Adolescents with disabilities and their families were often excluded from their communities, which worsened during humanitarian emergencies. Stigma and shame meant that adolescents with disabilities and their families were often isolated at home. They were subject to abuse if they went outside [25,30,31,37]. Parents of adolescent girls with disabilities in conflict-affected contexts reported that extended family were reluctant to be associated with them because of fears that this would negatively affect the marriage options of family members [25]. In Turkana, Kenya, everyone was struggling to meet their own needs which meant there was a lack of support for families of adolescents with disabilities [30].

Refugee adolescents with disabilities, especially girls, and those who had hearing, intellectual or severe physical impairments, faced especially high levels of exclusion from community services. They were more likely to be socially isolated than adolescents with disabilities in other non-humanitarian contexts, partly because humanitarian budgets were over-stretched and this prevented outreach efforts [37]. They were excluded from programming and wider participation in community life because of misconceptions about their capacities and identity, discriminatory attitudes, and parental concern about their safety [37,48]. They also experienced communication and environmental barriers to participation [48]. Difficult terrain, lack of accessible infrastructure and transportation, and lack of assistive devices made it harder for them to move around [30,37]. Some adolescents with disabilities in such contexts participated in community life online [25]. Confidence, proactivity, and emotional capacity of the parents of adolescents with disabilities affected the adolescents’ ability to thrive [25].

#### Isolation during the pandemic

The pandemic exacerbated environmental and attitudinal barriers to participating in community life. This contributed to the isolation of adolescents with disabilities, and some experienced heightened levels of disability stigma and discrimination [34,35,38,39,47]. Adolescents with disabilities were often isolated during the pandemic and were not able to leave their homes as much as non-disabled adolescents. This restricted movement and activity meant that they were more exposed to increased tensions and frayed family relationships [22,34,38,39,44]. This was difficult and they missed having fun and supportive friendship [22,26]. They were less likely to play than adolescents without disabilities, and they were less likely to interact with friends, in-person or online [39,42]. This isolation was partly due to their lower levels of ownership of devices with internet access [39]. However, being stuck inside during lockdown had a positive effect on family relationships for some adolescents with disabilities. For some, their fathers became more involved in their lives [22,38].

### Inclusion in emergency response

Few studies in humanitarian or emergency contexts examined the inclusion of adolescents with disabilities in emergency responses. During the pandemic, many governments took no specific measures to protect the life, health, and safety of persons with disabilities [22,32,35,42,45,47]. Poverty, mobility constraints, and lack of adapted activities also meant it was harder for adolescents with disabilities to volunteer in the pandemic response [39].

In conflict contexts, lack of data to identify adolescents with disabilities meant that humanitarian agencies could not always ensure an inclusive response [31]. This resulted in lack of access to aid, assistive devices, services and facilities in displacement camps [31]. In Turkana, Kenya, stigma prevented adolescents with disabilities from accessing food aid as some families were embarrassed to be with them in public [30]. It was logistically difficult for families of adolescents with disabilities to access food aid – either the environment was inaccessible or they could not leave their children unattended [30]. Food for work programmes often excluded caregivers by not accounting for their childcare needs [30].

## Discussion

We have described the experiences of adolescents with disabilities in humanitarian emergencies including disease outbreaks (e.g. COVID-19) natural disasters such as earthquakes and conflict in LMICs, and mapped the methods used. We found that humanitarian emergencies usually increased the vulnerabilities of adolescents with disabilities, and studies reported on effects across health, education, livelihoods, social protection, and community participation domains.

### Research methods

There were surprisingly few studies which documented adolescents with disabilities’ experiences directly or specifically, and many studies combined data from 1) disabled and non-disabled adolescents, 2) disabled adults and adolescents with disabilities, or 3) adolescents with disabilities and caregivers. This made it challenging to understand the specifics of adolescents with disabilities experience in LMICs.

Only seven of the thirty studies included in our review used participatory methods. This could partly be because some studies did not use in-person data collection. Using participatory methods with adolescents with disabilities helps to ensure that data collection is non-discriminatory, age-appropriate and inclusive, and that it recognises the rights and competencies of adolescents with disabilities [52]. Other benefits have been identified such as addressing intersectional power imbalances [53] ensuring focus on issues of relevance to adolescents with disabilities and enabling the development of sensitive tools and research methods. This can improve the quality and authenticity of the data [14,54] and improve knowledge transfer [13]. Participation can also benefit adolescents with disabilities directly through skills development, increased self-confidence and expanded social networks [54], and can challenge infantilisation of adolescents with disabilities [55]. Despite these advantages, researchers and NGOs can be reluctant to involve adolescents with disabilities in research. There is a lack of training and guidance on best practices [53] and they may underestimate adolescents with disabilities capacity to participate [14]. Toolkits (for example see Małachowska et al. [56]) and capacity building resources specifically for research with adolescents with disabilities would be useful for researchers and NGOs in order to promote the inclusion of adolescents with disabilities in research, stimulate ideas and develop confidence.

### Reporting of methods

Much of the literature in our review was grey literature, and most of the peer-reviewed literature was about humanitarian contexts. The lack of methodological rigour and unclear reporting of sampling made it difficult to assess quality, generalisability and often resulted in the development of broad recommendations (see also Hillgrove et al. [1]). When we presented our findings and draft implications to our advisory committee, they agreed that clearer reporting of methods would help NGOs learn about and use rigorous and innovative approaches. The bias towards grey literature may reflect the time-lag taken for the peer-review process, and the fact that many organisations used their research for advocacy and immediate response preparation. Other barriers may be financial, language related or a lack of knowledge about how to write about methods which could discourage submission to peer-reviewed journals. Our advisory committee discussed their lack of awareness about which journals would be interested in reviewing and publishing studies from NGOs. They were of the view that partnership approaches between academics and NGOs may improve both research methods and reporting.

Findings from grey and peer-reviewed literature were powerful and show that it has been possible to elicit opinions directly from adolescents with disabilities even in very difficult contexts. There is potential for clinicians and practitioners to conduct research with adolescents with disabilities enabling their unique experiences to be integrated in policies and programmes. The effects of the pandemic and humanitarian emergencies were different across different contexts, and there is a need to develop the capacity of local organisations to conduct rigorous research which can inform local and international responses [2].

### Adolescents with disabilities’ experiences

The experiences of adolescents with disabilities across the different humanitarian emergencies were similar in several ways: barriers to accessing key services such as health and education; family poverty; increased social isolation; and a lack of consideration of their needs in emergency responses. This led to them having a worse experience than non-disabled adolescents. Often, the pandemic intensified the difficulties faced by adolescents with disabilities in conflict or displacement affected settings, but there were some contextual differences. For example, adolescents with disabilities’ access to education during COVID-19 was limited by barriers to home schooling, whereas in conflict, displacement or emergency contexts, schools were destroyed, and adolescents faced security issues while trying to access schools.

### Research gaps

In general, the low number of published studies indicate that there is a need for more rigorous and specific research from LMIC contexts about how adolescents with disabilities experience humanitarian emergencies. Few studies in our review examined the inclusion of adolescents with disabilities in emergency responses. Reviews of research on people with disabilities have also noted the lack of research that reports on inclusion of people with disabilities in decision-making about COVID-19 [57]. This may be because there is a lack of data which leads to a lack of consideration about the needs of adolescents with disabilities. It could also be related to existing stigma and assumptions about the capacity of people with disabilities to participate in emergency responses. People with disabilities are under-represented in planning and decision-making structures which respond to humanitarian emergencies [58,59].

## Limitations

The size of our team enabled us to review a large amount of literature in a short time, but there may have been inconsistency across multiple researchers in charting, with some researchers reporting more detail than others. Given our time constraints, and our focus on the broad impacts of COVID-19 and humanitarian emergencies on adolescents with disabilities, we did not include Embase, Medline/PubMed, and CINAHL databases in our search strategy. We used the non-specific search terms of ‘disease outbreak’ and ‘natural disaster’ and were only specific in searching for COVID-19. This may have biased our search, as results from these categories only revealed studies on COVID-19 and earthquakes. We did not disaggregate studies by disability type or severity of impairment in our review because the literature did not disaggregate this systematically. This is an important consideration when analysing intersectional marginalization because of humanitarian emergencies.

## Conclusions

Humanitarian emergencies such as COVID-19 exacerbate pre-existing inequalities which means that adolescents with disabilities are amongst the most disadvantaged. Our review shows that it has been possible to conduct research with adolescents with disabilities to explore their experiences of humanitarian emergencies, and these experiences were different from those of non-disabled adolescents. However, inconsistent reporting of methods and aggregation of findings with disabled and non-disabled adults and non-disabled adolescents affected the specificity of study findings. The low number of studies showed a clear need for more research with adolescents with disabilities in LMICs. The predominance of grey literature about adolescents with disabilities’ experiences demonstrates the need to develop capacity in disabled persons’ organizations and NGOs to conduct and report rigorous research. Working in partnerships can facilitate this.

## Paper context

COVID-19 and other humanitarian emergencies have exacerbated pre-existing challenges for adolescents with disabilities. Yet few studies report their experiences. This scoping review highlights the scarcity of literature and the importance of conducting research on and with adolescents with disabilities in humanitarian contexts. Disaggregated data are needed in order to understand their unique experiences in humanitarian emergencies. It is important to develop research capacity of organisations of people with disabilities to encourage rigour and clear reporting.
